# Could cooling dialysate improve inflammatory and nutritional status of hemodialysis patients?

**DOI:** 10.1186/s12882-023-03305-z

**Published:** 2023-08-25

**Authors:** Asmaa Elemshaty, Nagy Sayed-Ahmed, Abeer Mesbah, Mohammed Kamal Nassar

**Affiliations:** 1 Mansoura Nephrology & Dialysis Unit (MNDU), Department of Internal Medicine, Faculty of Medicine, Mansoura University, Mansoura, Egypt; 2 Department of Internal Medicine, Faculty of Medicine, Horus University, New Damietta, Egypt; 3 Clinical Pathology department, Faculty of Medicine, Mansoura University, Mansoura, Egypt

**Keywords:** Hemodialysis, Cooling dialysate, Malnutrition, Inflammation

## Abstract

**Background:**

It has been shown that dialysate cooling (lowering the dialysate temperature to 0.5 °C below central body temperature) reduces the incidence of intradialytic hypotension. Other influences on hemodialysis patients, however, have not been adequately investigated. The purpose of this study was to determine the impact of individualized dialysate cooling on nutritional and inflammatory parameters in chronic hemodialysis (HD) patients.

**Methods:**

Seventy HD patients were separated into two groups: group A: (control group) standard dialysate temperature was 37 °C, and group B: (intervention group) dialysate temperature was 0.5 °C below core body temperature. In addition to routine laboratory tests, blood pressure, anthropometric measurements, inflammatory markers, and the malnutrition inflammation score (MIS) were calculated.

**Results:**

After six months of dialysate cooling, intradialytic hypotension episodes were much less prevalent in the intervention group (p = 0.001). Serum ferritin, transferrin saturation (TSAT), high sensitive C-reactive protein (HS-CRP), and Interleukin-6 (IL-6) reduced following dialysate cooling, whereas serum albumin rose. In the control group, IL-6 dropped but serum ferritin, TSAT, albumin, and HS-CRP rose. In both groups, hemoglobin levels dropped, and erythrocyte sedimentation rate (ESR) rose, both groups’ midarm muscle circumference and MIS worsened.

**Conclusion:**

Cold dialysate decreased intradialytic hypotension with no significant improvement of the nutritional and inflammatory surrogates. However, more studies including larger number of patients with longer duration of follow up are required to adequately assess its effect on inflammation and nutrition in chronic hemodialysis patients.

## Background

Chronic kidney disease (CKD) is a worldwide public health issue [1, 2]. Dialysis patients have a tenfold greater relative risk of cardiovascular death than the general population [3]. Patients undergoing hemodialysis (HD) often have inflammation [4]. Malnutrition is a frequent finding in patients with CKD, affecting 18–75% of HD patients and 10–50% of peritoneal dialysis patients [5]. Chronic inflammation and malnutrition are well-known risk factors for cardiovascular diseases in HD patients [4, 6]. Inflammatory markers, such as C-reactive protein (CRP) and interleukin 6 (IL-6), are associated with protein-energy wasting [7] and cardiovascular morbidity and mortality in dialysis patients [8].

Cold dialysis uses dialysate 0.5 °C below core body temperature [9]. Dialysate cooling prevents intradialytic hypotension (IDH) [10, 11]. This is achieved by inducing vasoconstriction and activating the sympathetic nervous and therefore improving hemodynamic stability [12]. Cold dialysis reduces HD-induced brain damage by protecting the cerebral vascular beds from harmful perfusion [9]. In the heart, long-term cold dialysis improved resting ejection fraction and reduced left ventricular mass and end-diastolic volumes while preserving aortic distensibility, decreasing the risk for future cardiovascular events [13]. On the contrary, a recent multi-center, two-arm, open-label, cluster-randomized trial in Canada concluded that personalized cool dialysate has no effect on cardiovascular health when compared to standard dialysate temperature of 365 °C [14].

It is anticipated that cooling the dialysate will prevent IDH and contribute to hemodynamic stability during the course of dialysis sessions, thereby facilitating the extension of HD sessions for sufficient time to allow for improved dialysis quality, as frequent IDH was found to be associated with reduced uremic solutes clearance [15]. This improved hemodynamic stability may have an effect on inflammation and malnutrition, as the reduced dialysis adequacy with frequent IDH has been found to result in uremia and metabolic acidosis, which promotes inflammation and tissue resistance to multiple anabolic hormones and simultaneously increases the activity of catabolic corticosteroids [16].

It would be fascinating to study the influence of cooled dialysate dialysis on such dreadful conditions as inflammation and malnutrition. Unfortunately, there is a paucity of literature on this topic, with the majority of research focusing on the association between cooled dialysate and its impact on blood pressure [10, 11] and cardiovascular complications [13] .

## Methods

### Patients

In this open-label, randomized, controlled interventional study, seventy adult (age > 18) ESRD patients undergoing regular hemodialysis for more than six months in the Dialysis Unit of Mansoura University Hospitals, Egypt were recruited. Patients who had a recent active infection or hospitalization, an underlying malignancy, sever disability, amputated arms or legs, were over the age of 75 years or had decompensated organ failure, other than renal failure (e.g. decompensated hepatic or heart failure), were excluded. The enrolled patients were divided into two groups; intervention group (group A) (n = 30) were subjected to individualized cool dialysate (dialysate temperature 0.5 °C lower than core body temperature) and control group (group B) (n = 40) were subjected to standard dialysate temperature (dialysate temperature of 37 °C). The dialysis techniques used in the study were conventional hemodialysis and ultrafiltration guided by the clinical condition of the patient. The time and frequency of hemodialysis sessions were four hours per session and three sessions per week, and the type of dialysis membranes were Fresenius FX filters with surface area 1.8 m2 with semisynthetic membrane helixone and Allmed filters with semisynthetic polysulfone membrane with surface area ranging from 1.8 to 2.2 m^2^.

The enrolled patients were allocated to their groups by simple randomization technique without knowing their characteristics. The patients who were scheduled for dialysis on the weekdays Saturday, Monday and Wednesday were allocated to be the intervention group, while those who were scheduled on the weekdays Sunday, Tuesday and Thursday were allocated to be the control group. This method of randomization helped the attending staff of a certain day to stick to one method every time they look after the recruited patients of that day. The Institutional Research Board of the Faculty of Medicine, Mansoura University, approved the study protocol (code: MS.19.04.592). The study was explained to all patients and informed written consent was obtained from all of them before starting the study.

Patients’ demographic data, such as age and gender, were collected. In addition, clinical characteristics such as hemodialysis duration, original kidney disease, and the presence of diabetes and hypertension were recorded. Both systolic and diastolic blood pressure were measured and averaged for four random dialysis sessions at the beginning of the study, every two months then at the end of the study (baseline, 2nd month, 4th and 6th month). Intradialytic hypotension was defined according to Kidney Disease Outcomes Quality Initiative (KDOQI) as a decrease in systolic blood pressure (SBP) by ≥ 20 mmHg or a decrease in mean arterial pressure (MAP) by 10 mmHg from the predialysis value, accompanied by symptoms including abdominal discomfort, yawning, sighing, nausea, vomiting, muscle cramps, restlessness, dizziness, fainting, and anxiety [17]. The intradialytic hypotensive episodes were recorded during the whole duration of the study (6 months). The subsequent assessments were done twice (at the beginning of study (Baseline) and six months following randomization).

### Blood sampling and laboratory tests

Before the first HD session of the week, blood samples were collected from the arteriovenous fistula. All patients were subjected to pre-dialysis basic laboratory investigations (complete blood count (CBC), serum iron, total iron binding capacity (TIBC), transferrin saturation (TSAT), serum ferritin, serum calcium, phosphorus, parathyroid hormone (PTH), serum albumin, cholesterol) on the first session of the week in addition to erythrocyte sedimentation rate (ESR), IL-6 and high sensitive C-reactive protein (HS-CRP). The IL-6 was measured by the commercial kit IL-6 ELISA kit, catalogue number is E0079h, Wuhan EIAab Science Co., Ltd, Wuhan, China.

### Anthropometric measurements

After measuring body weight (kg) and height (m) after dialysis, the body mass index (BMI) was determined. Mid upper arm circumference (MAC) was measured twice, two weeks apart, using a flexible, inelastic measuring tape in the non-arteriovenous fistula arm, just at the midpoint of upper arm (i.e. between the acromion process of scapula and the olecranon process of ulna), in sitting position, with the average being recorded [18]. Twice, two weeks apart, the Triceps skin fold (TSF) thickness was measured in millimetres. It was measured at the midway between the scapular acromion process and the ulnar olecranon process [18]. Mid arm muscle circumference (MAMC) was estimated in centimeters using the following formula: MAMC = MAC– (3.14 x TSF thickness) [19].

### Malnutrition inflammation score

Malnutrition inflammation score (MIS) is comprised of ten elements, including weight change, dietary intake, gastrointestinal symptoms, functional ability, comorbidity, subcutaneous fat, and signs of muscle wasting, BMI, serum albumin, and TIBC. Each component receives a score between 0 (normal) and 3 (very severe). It is anticipated that the total of all 10 compartments will fall between 0 (well-nourished) and 30 (severely malnourished) [20].

Both anthropometric measurements and MIS assessments were done by the same observer.

### Sample size and statistical analysis

According to the study hypothesis, comparing the change in inflammatory and nutritional parameters, using the MIS which is considered as a valuable indicator of nutritional and inflammatory status in hemodialysis patients [20], in group A (cooling dialysate) vs. group B (control) would have a large effect size (Cohen’s d = 0.8). A large effect size means that research finding has practical significance [21]. Group sample sizes of 30 group A cases and 40 group B cases achieve 90.41% power to reject the null hypothesis of zero effect size when the population effect size is 0.80 and the significance level (α) is 0.050 using a two-sided two-sample equal-variance t-test. Sample size was calculated by using G*Power software (version 3.1.9.7).

SPSS (Statistical Package of Social Sciences) version 21 for Windows (SPSS, Inc) was used to conduct the statistical analysis. Number and percent were used to describe qualitative data (n, %). When applicable, the data was first evaluated for normality using the Shapiro-Wilk test or the Kolmogorov-Smirnov test. For normally distributed data, mean ± SD (standard deviation) was used, and for non-normally distributed data, median (interquartile range) was used. When comparing two groups with quantitative normally distributed data, the Independent-Samples T test and Paired-Samples T test were used, whereas when comparing two groups with quantitative non- normally distributed data, the Mann-Whitney test and Wilcoxon test were employed. When comparing qualitative data with a 2 × 2 table, the chi-square or Fisher’s exact test was applied. Univariate correlation analysis was carried out with the Pearson test for normally distributed data and the Spearman test for non-normally distributed variables. A statistically significant P value was less than 0.05.

## Results

The baseline demographic and clinical data, as well as the anthropometric measurements, revealed no statistically significant differences between the two research groups. Except for serum ferritin, which was considerably greater in group A, there were no other significant differences in the measured laboratory tests between the two groups (Table 1).

**Table 1:** comparison of baseline demographic and clinical data, anthropometric measurements and laboratory data between cooling and non-cooling groups

Item				Group A (n = 30)	Group B (n = 40)	*P*
Demographic and Clinical data						
Age (years)				51 ± 18.15	51.95 ± 14.65	0.80^*^
Gender		Male Female		16 (53.3) 14 (46.7)	23 (57.5) 17 (42.5)	0.72^#^
DM				4 (13.3)	7 (17.5)	0.44^#^
HTN				18 (60)	20 (50)	0.40^#^
Original disease	Diabetic nephropathy Hypertensive nephropathy Polycystic kidney disease Interstitial nephritis SLE Failed Transplantation Unknown Obstructive uropathy Chronic GN			2 (6.7) 14 (46.7) 0 2 (6.7) 0 0 10 (33.3) 2 (6.7) 0	3 (7.5) 21 (52.5) 1 (2.5) 2 (5) 1 (2.5) 1 (2.5) 9 (22.5) 1 (2.5) 1 (2.5)	0.79^#^
HD duration(years)				4.5 (3–7)	3.5 (2–7)	0.17^Ω^
BP			DBP SBP	139.8 ± 16.58 80.8 ± 7.43	137.7 ± 11.7 79.7 ± 6.29	0.54^*^ 0.51^*^
Anthropometric measurements						
BMI (kg/m^2^)				29.6 ± 6.73	28.6 ± 5.13	0.45^*^
Weight(kg)				79.5 ± 17.33	78.5 ± 15.86	0.8^*^
Height(m)				163.1 ± 9.86	165.9 ± 8.5	0.21^*^
MAC (cm)				29.6 ± 4.55	28.8 ± 4.66	0.49^*^
SFT (cm)				17.7 ± 7.35	17.9 ± 7.65	0.91^*^
MAMC (cm)				24.9 ± 2.91	24.5 ± 3.8	0.64^*^
MIS				3 (2–5)	4 (2.25–5.75)	0.20 ^Ω^
Laboratory data						
Calcium (mg/dl)				8.45 ± 1.1	8.1 ± 1.03	0.17*
Phosphorus (mg/dl)				5.27 ± 1.81	4.97 ± 1.07	0.48*
P.T.H (pg/ml)				583 (171–1129)	570 (332–1106)	0.48 ^Ω^
Albumin (g/dl)				3.85 ± 0.35	3.79 ± 0.36	0.47*
Cholesterol				158.9 ± 38.26	168.2 ± 43.87	0.35*
Hemoglobin (g/dl)				11.5 ± 2.26	10.9 ± 1.72	0.25^Ω^
Ferritin (ng/ml)				1157 (604–1338)	680 (443–932)	**0.02** ^Ω^
TSAT (%)				35 (29.5–55.5)	35.5 (27-44.75)	0.39 ^Ω^
ESR (mm\hr)				25 (15-31.25)	30 (10-44.75)	0.75 ^Ω^
HS-CRP (mg\l)				14.15 (5.88–21.08)	11.55 (4.90-18.58)	0.45 ^Ω^
IL-6 (pg\ml)				4.4 (3.85-5)	4.2 (3.32–4.8)	0.31 ^Ω^
KT/V				1.19 ± 0.32	1.17 ± 0.32	0.76 *

(BP) Blood Pressure, (SBP) Systolic Blood Pressure, (DBP) Diastolic Blood Pressure, (BMI) Body mass index, (MAC) Midarm circumference, (TSF) Triceps skin fold thickness(cm), (MAMC) Midarm muscle circumference, (MIS) Malnutrition inflammation score, PTH: Parathyroid hormone, HS-CRP: High sensitive CRP, IL-6: Interleukin-6, ESR: Erythrocyte sedimentation rate (ESR).^*^ 2-sample t test; ^#^ Chi-square; ^Ω^ Mann Whitney test

Regarding blood pressure measurements, there was no statistically significant difference between the two groups for measurements at baseline and the sixth month, nor for bouts of intradialytic hypotension at baseline. At the end of the study, however, more patients in the intervention group had no IDH events, and the overall number of episodes each month was lower in this group (Table 2).

**Table 2:** Serial pre dialytic blood pressure comparison (average measurements of 4 occasion per month) and IDH between the baseline and 6-month data between the two groups:

Item		Group A (n = 30)	Group B (n = 40)	*P*
Baseline BP	SBP DBP	139.8 ± 16.58 80.8 ± 7.43	137.7 ± 11.7 79.7 ± 6.29	0.54^*^ 0.51^*^
6th month BP	SBP BBP	139.6 ± 13.32 79.8 ± 7.24	135.1 ± 11.68 79.2 ± 5.25	0.13^*^ 0.69^*^
IDH at Baseline	No episode 1 episode 2 episodes 3 episodes	14(46.7) 6(20) 6(20) 4(13.3)	18(45) 12(30) 5(12.5) 5(12.5)	0.729^**^
IDH after 6 months	No episode 1 episode 2 episodes 3 episodes	20(66.7) 9(30) 1(3.3) 0	17(42.5) 11(27.5) 8(20) 4(10)	**0.035** ^**^
p (Chi-square)		**0.001**	0.527	
Total number of episodes in a month (%)^a^	**Before**	30 (7.69%)	37 (7.12%)	0.742^#^
	**After**	11 (2.82%)	39 (7.50%)	**0.002** ^#^
P		**0.002** ^#^	0.0.812^#^	

SBP: systolic blood pressure, DBP: diastolic blood pressure, IDG: intradialytic hyotension

^*^Analyzed by 2-sample t test; ^**^analyzed by Wilcoxon test; ^#^N-1 Chi-squared test as recommended by Campbell (2007) and Richardson (2011)

^**a**^ Of the total number of sessions during the same month (n = 390 for group A, and 520 for group B)

In the group receiving cooled dialysate (group A), there was a statistically significant drop in serum ferritin, TSAT, HS-CRP, and Interleukin-6 after 6 months of cooling, but serum albumin rose, relative to the baseline values. The 6-months assessments of the group of patients who were not subjected to dialysate cooling (group B) revealed a substantial drop in IL-6, but no significant change in serum ferritin and TSAT, and a significant rise in serum albumin and HS-CRP, relative to their baseline values. Hemoglobin level decreased significantly and ESR increased significantly after 6 months of observation in both group A and group B patients. Unfortunately, both MAMC and MIS worsened after 6 months in both groups of patients (Table 3).

**Table 3:** Anthropometric measurements and laboratory data comparison between baseline and 6 months measures for the two groups:

Item	Group with cooled dialysate (n = 30)			Group without cooled dialysate (n = 40)		
	Baseline	6 months	P	Baseline	6 Months	P
Anthropometric measurements						
BMI (kg/m^2^)	29.6 ± 6.73	28.9 ± 6.93	0.20	28.6 ± 5.13	28.6 ± 5.1	0.92
Weight(kg)	80.3 (60.6–86.3)	80 (63.6–90)	0.58	79.4 (64.8–95.5)	80 (65.5–96)	0.49
MAC (cm)	29.6 ± 4.55	29.9 ± 5.34	0.82	28.8 ± 4.66	28.7 ± 4.33	0.93
SFT (cm)	17.7 ± 7.35	21.7 ± 10.86	0.06	17.9 ± 7.65	15.3 ± 8.66	0.11
MAMC (cm)	24.9 ± 2.91	23.1 ± 3.62	0.01	24.5 ± 3.8	24.08 ± 4.01	0.54
MIS	3 (2–5)	7 (5–10)	**< 0.001**	4 (2.25–5.75)	7.5 (5.25–9.75)	**< 0.001**
Laboratory data						
Calcium (mg/dl)	8.45 ± 1.1	8.51 ± 1.1	0.59	8.1 ± 1.03	8.51 ± 1.41	0.08
Phosphorus (mg/dl)	5.27 ± 1.81	5.96 ± 1.74	0.08	4.97 ± 1.07	5.55 ± 2.29	0.17
P.T.H (pg/ml)	583 (171–1129)	634 (257–1081)	0.32	570 (332–1106)	600 (361–971)	0.32
Albumin (g/dl)	3.85 ± 0.35	4.75 ± 0.63	**< 0.001**	3.79 ± 0.36	4.65 ± 0.62	**< 0.001**
Cholesterol (mg/dl)	158.9 ± 38.26	140.4 ± 46.37	0.052	168.2 ± 43.87	125.7 ± 28.21	**< 0.001**
Hemoglobin (g/dl)	11.5 ± 2.26	9.6 ± 1.45	**< 0.001**	10.9 ± 1.72	9.96 ± 1.57	**< 0.001**
Ferritin (ng/ml)	1157 (604–1338)	662 (430–1131)	**< 0.001**	680 (443–932)	536 (381–755)	0.12
TSAT (%)	35 (29.5–55.5)	33 (24.75-38)	**0.004**	35.5 (27-44.75)	30 (21.5–38)	0.07
ESR (mm\hr)	25 (15-31.25)	40.5 (28-66.25)	**< 0.001**	30 (10-44.75)	52.5 (22-77.5)	**< 0.001**
HS-CRP (mg\l)	14.150 (5.875–21.075)	12.100 (6.075–12.125)	**0.002**	11.55 (4.9-18.575)	12.1 (7.45–12.1)	**0.02**
IL-6 (pg\ml)	4.4 (3.85-5)	2.2 (0.8–3.92)	**< 0.001**	4.2 (3.32–4.8)	0.8 (0.7–0.9)	**< 0.001**
KT/V	1.19 ± 0.32	1.17 ± 0.63	0.87	1.17 ± 0.32	1.3 ± 0.47	0.08

(PTH) Parathyroid hormone, (TSAT) Transferrin saturation, (HSCRP) High sensitive CRP, (IL-6) Interleukin-6, (ESR)Erythrocyte sedimentation rate, (BMI) Body mass index, (MAC) Midarm circumference, (TSF) Triceps skin fold thickness(cm), (MAMC) Midarm muscle circumference, (MIS) Malnutrition inflammation score

Regarding the adverse effects of cooling, more patients in the intervention group experienced shivering and discomfort as a result of cooling. However, the clinical impact of these side effects was minor, and no patient withdrew from the study (Table 4).

**Table 4:** cooling side effects in both groups

Item		Group A (n = 30)	Group B (n = 40)	*p*
Cooling side effects	No Shivering discomfort	21(70) 4(13.3) 5(16.7)	39(97.5) 1(2.5) 0	**0.004** ^**^

^**^chi square test

## Discussion

In the current study, some inflammatory markers and the prevalence of intradialytic hypotensive episodes decreased after six months of dialysate cooling, although midarm circumference decreased and MIS deteriorated.

Using the Malnutrition-Inflammation Score to examine inflammation and malnutrition is commonly practiced [22]. More than 90% of patients in the current study had a relatively mild degree of malnutrition and inflammation (MIS score < 9) at baseline. As its data did not demonstrate regression after the intervention, MIS did not improve after dialysate cooling; in fact, more than 25% of patients in the current research who were treated to dialysate cooling obtained a MIS below 9, indicating a worsening of their malnutrition/inflammation status. Similarly, the control group’s MIS rose in follow-up observations. These findings indicate that cooling of dialysate does not affect the nutritional and inflammatory status of the patients. During the longitudinal part of their study, Beberashvili and colleagues found that MIS exhibited a decreasing trend over time [23]. In a 12-month Chinese study involving 59 peritoneal dialysis patients, only one-third of the patients exhibited worsening MIS [24]. These contradictory results can be explained by the shorter duration of follow-up in the current study, the different dialysis modality in the Chinese study and the different patient population, as our patients were younger and of a different ethnic group. Probably the patient in the present study could have been exposed to different perpetuating factors that we were not controlled for in the study and might have an impact on different studies value independently. In both the cooling and non-cooling groups, anemia and iron status worsened, which may have led to an increase in 6-month MIS levels.

Serum albumin level, a commonly used marker of nutritional status in ESRD patients [25, 26] that has been shown to be a strong predictor of morbidity and mortality in dialysis patients [26–28], showed a statistically significant improvement after 6 months of observation in the present study. The fact that both groups improved in albumin prevents a conclusive conclusion concerning dialysate cooling’s influence on plasma albumin. Albumin improvement can’t be firmly linked to dialysate cooling. The patients may have felt like they were the center of medical care, which may have improved their nutrition.

Inflammatory markers are the product of interaction between noxious agents and the immune system. They could include, among others, granulocytic reaction, increased ESR, CRP and ferritin, and certain interleukins [29]. Serum ferritin, a marker originally used to reflect body iron storage [30, 31] and to monitor iron therapy in CKD patients [32], has been identified as a surrogate marker of inflammation. In the current work, it was markedly elevated at the baseline in the intervention group relative to its reference range, reflecting a degree of inflammation in these patients, having observed that the median TSAT value was below the limit diagnosing iron overload states. Despite the significant decrease in the intervention group, there was no significant difference between the two groups in serum ferritin levels at the end of the study. Parallel to the change of serum ferritin, HS-CRP showed the same tendency of being significantly decreased after the observed duration of dialysate cooling, while it did not show the same tendency following the observation period without application of the same intervention. Even though the difference was statistically significant, it was not numerically significant.

Interleukin-6 is an important cytokine that has been increasingly recognized as a central regulator of the inflammatory process and is known to play a key role in the induction of the immune effector mechanisms and acute-phase responses. Unlike other cytokines, IL-6 encompasses both endocrine and paracrine effects [33]. In the current study, interleukin − 6 was observed to be statistically significantly decreased following the studied period of dialysate cooling. The control group showed similar or even greater IL-6 improvement. These findings hamper the inflammatory suppressive role of individualized dialysate cooling. Other studies involving hemodialysis [34] and peritoneal dialysis [35] patients found a tendency for IL-6 levels to remain stable over 3 and 1 years of follow-up, respectively. The shorter duration of follow-up in the present study, as well as the distinct dialysis modalities of extended hemodialysis and peritoneal dialysis, may account for these contradictory findings. The discrepant changes of IL-6 and CRP, as well as of MIS and IL-6 can be explained as IL-6 is believed to be a vulnerable molecule and is subjected to change by multitude of various trivial factors like intercurrent infection, allergic reaction during dialysis, intake of non-steroidal anti-inflammatory drugs and others, many of these were not controlled in the present study. Moreover, the decrease in IL-6 measurements could be a regression to the mean and not a true decrease. This must be confirmed by serial cytokine measurements over an extended period of follow-up.

As stated previously, it has been seen frequently that dialysate cooling inhibits IDH [10, 11, 36]. In accordance with this, the current study showed improvement in IDH during dialysate cooling. Similarly, a meta-analysis of 26 randomized controlled trials with 484 patients indicated that reducing dialysate temperature reduced IDH by 70% and elevated intradialytic MAP by 12 mmHg [37]. Recently [38], a study tested 62 dialysis patients and found that cold dialysate stabilized blood pressure and reduced IDH. The latter authors concluded that reducing dialysate temperature from 36.5 to 35 °C leads to hemodynamic stability. In contrast to these results, the recent multicenter open-label MYTEMP study involving 15,413 hemodialysis patients found no significant between-group differences in the risk of intradialytic hypotension [14]. This discrepancy can be explained by the larger number of patients in the latter study and the distinct definitions used to define intradialytic hypotension. The main measure for definition of intradialytic hypotension in this study was (i) nadir systolic blood pressure < 90 mm Hg anytime during a hemodialysis treatment when the value prior to starting the treatment was ≥ 90 mm Hg, or (ii) drop in systolic blood pressure ≥ 30 mm Hg anytime during a hemodialysis treatment from the value before starting the treatment [14].

However, dialysis based on cooled dialysate is not totally devoid of some side effects; the most commonly reported side effects are related to cold sensation, while some studies have also reported incidences of shivering [39]. In the current study, cooled dialysate infrequently induced discomfort and shivering; a finding that was previously described in previous studies [13, 40–42]. No other serious disadvantages of cold dialysis have been reported in the literature; even in the long-term MyTEMP study in which more patients in the cooler dialysate group reported feeling uncomfortably chilled during dialysis than those in the standard temperature dialysate group [14].

This study had limitations. First, a relatively small number of patients were studied. Second, the short duration of the study. Third, failure to control the trivial events that can affect the levels of inflammatory markers and the malnutrition inflammation score such as like intercurrent infection, allergic reaction during dialysis, intake of non-steroidal anti-inflammatory drugs. However, assessing the effect of cooled dialysis on inflammation and nutritional status in this specific group of patients is considered as a strength point in the current study.

## Conclusion

Cool dialysate for HD patients is safe and feasible. It also protected the patient from intra-dialytic hypotension, although it was not associated with better nutrition or inflammation. Further research with more patients and longer durations is needed to fully elucidate this issue.

## Data Availability

All data analyzed during the current study available from the corresponding author on reasonable request.

## Abbreviations

CKD: Chronic kidney disease
ESRD: End-stage renal disease
HD: Hemodialysis
CRP: C-reactive protein
IL-6: Interleukin 6
IDH: Intradialytic hypotension
CBC: Complete blood count
TIBC: Total iron binding capacity
TSAT: Transferrin saturation
PTH: Parathyroid hormone
ESR: Erythrocyte sedimentation rate
HS-CRP: High sensitive C-reactive protein
BMI: Body mass index
MAC: Mid upper arm circumference
TSF: Triceps skin fold
MAMC: Mid arm muscle circumference
MIS: Malnutrition inflammation score
